# Association between blood lead level and blood pressure: An occupational population-based study in Jiangsu province, China

**DOI:** 10.1371/journal.pone.0200289

**Published:** 2018-07-06

**Authors:** Lei Han, Xiuxia Wang, Ruhui Han, Ming Xu, Yuan Zhao, Qianqian Gao, Huanxi Shen, Hengdong Zhang

**Affiliations:** 1 Institute of Occupational Disease Prevention, Jiangsu Provincial Center for Disease Control and Prevention, Nanjing, China; 2 The Affiliated Guangji Hospital of Soochow University, Suzhou, China; 3 Department of Infection Management, the First Affiliated Hospital of Soochow University, Suzhou, China; 4 Kunshan Municipal Centre for Disease Prevention and Control, Kunshan, China; Shanghai Institute of Hypertension, CHINA

## Abstract

Studies about the association between lead exposure and the elevation of blood pressure and risk of hypertension are varied, while available data on blood lead levels (BLL) in workers with lead-exposure are scarce. This research aimed to evaluate associations between BLL and blood pressure in an occupational population-based study in Jiangsu province, China. We enrolled 21,688 workers in this study. Information on socioeconomic and occupational background was obtained with face-to-face interviews. BLL, systolic blood pressure (SBP) and diastolic blood pressure (DBP) were measured, and hypertension status was confirmed. We found that workers in mini-factories had the highest average BLL (20.3 μg/dL; 95% CI, 19.0–21.6 μg/dL) for overall participants. The employees in private factories had higher BLL (9.6 μg/dL; 95% CI, 9.5–9.8 μg/dL). However, BLL was much lower (4.0 μg/dL; 95%CI, 3.7–4.2 μg/dL) in state-owned factories. Participants working in the electrical machinery and equipment manufacturing industry had higher BLL (9.1 μg/dL; 95% CI, 9.0–9.3μg/dL). Compared to those workers with ≤ 4.6 μg/dL BLL, workers with > 17.5 μg/dL BLL presented 1.34 mmHg and 0.70 mmHg average difference in SBP and DBP, respectively. The adjusted OR for hypertension was 1.11 (95%CI, 1.08–1.15) compared to the workers with > 17.5 μg/dL BLL and to those with ≤ 4.6 μg/dL BLL. In summary, we found that BLL was positively associated with SBP and DBP and with the morbidity of hypertension in occupational populations with a high concentration of lead exposure. It is important to formulate new standards of blood lead levels to screen for elevated lead exposure. In addition, a series of new systems of risk assessment should be established to further reduce and prevent lead exposure.

## Introduction

Lead (Pb) is a pervasive occupational toxicant in many industries, such as the electrical equipment manufacturing industry and mining industry. The toxic effects of lead have been documented since the ancient times of Greece and China[1]. Inhalation, ingestion and dermal absorption are the two main routes of exposure to lead [2], and inhalation is the primary route of occupational exposure. It has been demonstrated that acute and chronic occupational exposure to lead may lead to progressive health effects in several human organs and systems including the nervous, haematopoietic, and cardiovascular systems, as well as the kidney and bones[3]. Based on data from 2015, the Institute for Health Metrics and Evaluation has estimated that lead exposure accounted for 494,550 deaths and 9.3 million disability adjusted life years (DALYs) due to long-term effects on health, with the highest burden in developing regions[4]. Even though the environmental lead concentration has steadily decreased in recent decades, higher concentrations of lead were still found in some populations occupationally exposed to lead[5].

The current Chinese standard for BLL is 40 μg/dL; exceeding this causes employees to be considered subjects of observation and requires that their blood lead concentration be monitored every 3~6 months[6]. However, according to the US National Toxicology Program, even a much lower BLL (<10 μg/dL) could cause adverse changes in kidney function, impair neurocognitive function and increase the risk of hypertension[7].

Hypertension is a heterogeneous disorder whose pathogenesis is not completely clarified [8]. As a major cardiovascular risk factor, hypertension results from the interplay of environmental and genetic factors [9]. Common risk factors include age, obesity, elevated sodium intake, smoking, alcohol consumption, kidney disease, and a family history of hypertension. Exposure to environmental chemicals, such as lead, is emerging as a potential risk factor for hypertension [10]. Several studies have reported that increased blood pressure, which is associated with the onset of cardiovascular diseases, has been linked to chronic lead exposure [11,12,13,14,15]. However, the mechanism of how lead increases blood pressure and causes hypertension is complex and poorly investigated. The probable mechanisms that cause hypertension due to long-term lead exposure may include physiological changes of the muscular and endothelial layers induced by disturbance in the renin-angiotensin-aldosterone system, abnormalities in the kallikrein-kinin system, stimulation of the sympathetic system and hyperreactivity of the vascular epithelial due to elevated production of catecholamines and reactive oxygen species [16]. The results of studies about population were varied. Some studies found increased blood pressure and increased risk of hypertension with high BLL[15,17,18], while the others found weak or no association[19,20].

The present study was undertaken to estimate the BLL and to assess the hypertension attributable to lead toxicity among industrial workers with lead exposure in Jiangsu, China.

## Materials and methods

### Study population

This study was conducted in 21688 workers who were exposed to lead in the workplace in Jiangsu province, China. The study population included adult workers aged 18 years or older whose duration of lead exposure was at least one year. Information on socioeconomic and occupational background was obtained by face-to-face interviews. The database included general demographic details and work-history records with the duration of lead exposure. Hypertension in participants was defined by the 2010 Chinese guidelines for the prevention and treatment of hypertension[21] (systolic blood pressure ≥140 mmHg and/or diastolic blood pressure ≥90 mmHg on at least two different visits) and self-reported use of antihypertensive medication. The study protocol was approved by the institutional review board of the Jiangsu Provincial Centres for Disease Control and Prevention, and written informed consent was obtained from all participants.

### Measurements

The measurements were taken according to the 2010 Chinese guidelines for the prevention and treatment of hypertension by using a mercurial sphygmomanometer. Measurements were obtained from both arms and two measurements were performed with a 1-2-minute interval between them, with the participant in a sitting position. The mean of the two blood pressure measurements was considered as the most accurate blood pressure and was used for statistical analyses.

Whole blood specimens of 5 ml for lead examination were collected from the cubital vein after an overnight fast of at least 8 h. Blood lead levels were determined by atomic absorption spectrometry (BH2200, China). Blood samples for analyses were deproteinised using 0.8 M nitric acid mixed with blood at a 4:1 ratio to determine the blood lead level. All samples were run in duplicate. The detection limit of lead was 0.3 μg/dL, and the precision of the results, expressed as the coefficient of variation, was 4.4%.

### Statistical analysis

For statistical analysis, the software SPSS 20.0 (IBM Corporation, Armonk, NY, USA) was used to perform descriptive and inferential statistical tests. Two-sided *P* values < 0.05 were considered significant. Blood lead levels were left skewed and log transformed for analysis. Multiple linear regression analysis was used to examine associations of blood lead with blood pressure. Multiple logistic regression analysis was used to evaluate the risk of hypertension by blood lead levels. Pearson correlation analysis was taken to verify the correlations between blood pressure and blood lead levels.

## Results

The overall geometric mean of BLL was 8.8 μg/dL (95% CI, 8.7–8.9), and the overall median was 10.9 μg/dL. BLL were higher in men (geometric mean was 9.0 μg/dL), in older participants (geometric mean was 13.1 μg/dL), in participants with longer lead exposure history (geometric mean was 11.7 μg/dL and 10.8 for 2–4 years and >4 years, respectively) (Table 1).

**Table 1:** Blood lead levels (μg/dL) by participant characteristics.

Characteristic	Overall				Male				Female			
	n (%)	Median (Q1-Q3)	Means (95% CI)	*P*	n (%)	Median (Q1-Q3)	Means (95% CI)	*P*	n (%)	Median (Q1-Q3)	Means (95% CI)	*P*
	21688 (100)	10.9 (4.6–17.5)	8.8 (8.7, 8.9)		13161 (100.0)	11.0 (4.6–18.2)	9.0 (8.8, 9.2)		8527 (100.0)	10.2 (4.6–16.2)	8.5 (8.3, 8.7)	
Age				< 0.001				< 0.001				< 0.001
≤ 30	6239 (28.8)	6.8 (2.8–13.6)	6.2 (6.0, 6.4)		4025 (30.6)	6.9 (2.9–13.6)	6.3 (6.1, 6.5)		2214 (26.0)	6.5 (2.7–13.6)	5.9 (5.7, 6.2)	
31–45	10690 (49.3)	11.0 (5.0–18.1)	9.0 (8.9, 9.2)		6135 (46.6)	11.1 (5.0–19.0)	9.2 (9.0, 9.5)		4555 (53.4)	11.0 (5.0–17.0)	8.8 (8.5, 9.0)	
≥ 46	4759 (21.9)	13.6 (8.1–25.2)	13.1 (12.7, 12.4)		3001 (22.8)	13.6 (8.4–26.5)	13.7 (13.2, 14.1)		1758 (20.6)	13.6 (7.6–22.8)	12.1 (11.6, 12.6)	
Exposure years				< 0.001				< 0.001				< 0.001
< 2	9626 (44.4)	7.3 (2.6–13.6)	6.4 (6.3, 6. 6)		6294 (47.8)	7.2 (2.6–13.6)	6.3 (6.2, 6.5)		3332 (39.1)	7.9 (2.6–13.6)	6.6 (6.4, 6.8)	
2–4	6304 (29.1)	13.6 (7.5–21.0)	11.7 (11.5, 12.0)		3492 (26.5)	13.6 (8.0–23.0)	12.7 (12.4, 13.1)		2812 (33.0)	13.6 (6.7–19.2)	10.6 (10.3, 11.0)	
> 4	5758 (26.5)	13.0 (6.2–20.4)	10.8 (10.6, 11.1)		3375 (25.6)	13.6 (7.0–23.0)	12.1 (11.8, 12.5)		2383 (27.9)	9.7 (5.3–16.8)	9.2 (8.8, 9.5)	
SBP				< 0.001				< 0.001				< 0.001
< 140 mm Hg	18852 (86.9)	10.0 (4.4–17.0)	8.5 (8.4, 8.7)		10988 (83.5)	10.0 (4.4–17.6)	8.7 (8.5, 8.9)		7864 (39.1)	10.0 (4.4–15.9)	8.3 (8.1, 8.5)	
≥ 140 mm Hg	2836 (13.1)	13.6 (6.8–21.4)	10.8 (10.4, 11.2)		2173 (16.5)	13.6 (6.8–21.5)	10.8 (10.4,11.3)		663 (7.8)	13.6 (6.9–21.3)	10.7 (9.9, 11.5)	
DBP				< 0.001				< 0.001				< 0.001
< 90 mm Hg	18340 (84.6)	10.0 (4.4–16.5)	8.4 (8.3, 8.6)		10608 (80.6)	10.0 (4.3–17.0)	8.6 (8.4, 8.7)		7732 (90.7)	10.0 (4.4–15.5)	8.3 (8.1, 8.5)	
≥ 90 mm Hg	3348 (15.4)	13.6 (6.7–22.8)	10.9 (10. 6, 11.3)		2553 (19.4)	13.6 (6.8–21.5)	11.0 (10.6, 11.5)		795 (9.3)	13.6 (6.7–22.6)	10.7 (9.9, 11.5)	
Hypertension				< 0.001				< 0.001				< 0.001
No	17578 (81.0)	9.8 (4.3–16.3)	8.4 (8.3, 8.5)		10069 (76.5)	9.8 (4.3–17.0)	8.5 (8.3, 8.7)		7509 (88.1)	9.8 (4.4–15.4)	8.2 (8.0, 8.4)	
Yes	4110 (19.0)	13.6 (6.7–22.3)	10.8 (10.4,11.1)		3092 (23.5)	13.6 (6.6–22.5)	10.8 (10.4,11.2)		1018 (11.9)	13.6 (6.7–22.0)	10.6 (10.0, 11.3)	
Factory scale				< 0.001				< 0.001				< 0.001
mini	437 (2.0)	23.1 (13.7–34.5)	20.3 (19.0, 21.6)		298 (2.3)	24.3 (13.6–36.4)	20.8 (19.3, 22.5)		139 (1.6)	21.4 (15.1–29.1)	19.1 (17.2, 21.2)	
small	3341 (15.4)	13.1 (5.0–18.6)	9.7 (9.4, 10.0)		1948 (14.8)	13.6 (5.6–19.6)	10.6 (10.1, 11.0)		1393 (16.3)	11.9 (4.2–15.8)	8.6 (8.1, 9.0)	
middle	11158 (51.4)	11.6 (6.5–17.7)	10.4 (10.2, 10.6)		6846 (52.0)	12.0 (6.6–19.0)	11.0 (10.7, 11.2)		4312 (50.6)	11.0 (6.4–15.0)	9.6 (9.4, 9.8)	
large	6752 (31.1)	6.8 (2.0–14.4)	6.0 (5.8, 6.2)		4096 (30.9)	6.4 (1.5–13.6)	5.6 (5.4, 5.8)		2683 (31.5)	7.4 (2.6–16.7)	6.6 (6.3, 6.9)	
Economic type				< 0.001				< 0.001				< 0.001
State-owned	840 (3.9)	3.6 (1.9–7.4)	4.0 (3.7, 4.2)		504 (3.8)	4.9 (2.3–9.3)	5.2 (4.7, 5.6)		336 (3.9)	2.5 (1.5–4.1)	2.7 (2.4, 2.9)	
Collectively-owned	2396 (11.0)	7.5 (6.2–8.9)	7.6 (7.5, 7.7)		1547 (11.8)	7.5 (6.2–8.8)	7.6 (7.5, 7.8)		849 (10.0)	7.6 (6.2–8.9)	7.6 (7.4, 7.8)	
Privately-owned	16741 (77.2)	13.6 (4.6–21.4)	9.6 (9.5, 9.8)		10221 (77.7)	13.6 (4.2–22.1)	9.7 (9.4, 9. 9)		6520 (76.5)	13.0 (5.2–20.5)	9.6 (9.4, 9.8)	
Foreign-owned	1711 (5.4)	7.6 (4.0–13.6)	6.5 (6.2, 6.7)		889 (6.8)	11.1 (4.5–13.6)	7.4 (7.0, 7.7)		822 (9.6)	6.0 (3.3–13.6)	5.6 (5.3, 6.0)	
Industry category				< 0.001				< 0.001				< 0.001
EM	17337 (79.9)	10.8 (4.7–20.0)	9.1 (9.0, 9.3)		10576 (80.4)	10.2 (4.3–20.7)	9.0 (8. 8, 9.1)		6761 (79.3)	11.2 (5.3–19.6)	9.4 (9.1, 9.6)	
CCOE	1317 (6.1)	7.8 (4.0–13.6)	6. 8 (6.5, 7.1)		651 (4.9)	13.6 (5.5–13.6)	7.9 (7.4, 8.3)		666 (7.8)	6.4 (3.0–13.6)	5.9 (5.5, 6.3)	
Others	3034 (14.0)	13.2 (4.6–13.6)	8.0 (7.7, 8.3)		1934 (14.7)	13.6 (5.7–13.6)	9.6 (9.3, 10.0)		1100 (12.9)	6.5(2.9–13.6)	5.7 (5.4, 6.1)	

SBP: systolic blood pressure; DBP: diastolic blood pressure; EM: electrical machinery and equipment manufacturing industry; CCOE: computer, communications and other electronic equipment manufacturing industry.

Among the 21688 participants in our study, 4110 (19.0%) were classified as hypertensive. A total of 2074 participants had both systolic and diastolic hypertension, 762 participants had only systolic hypertension, and 1274 participants had diastolic hypertension. Participants with hypertension had higher BLL (10.8 μg/dL; 95% CI, 10.4–11.1 μg/dL) than those people without hypertension (8.4 μg/dL; 95% CI, 8.3–8.5 μg/dL). The correlation between systolic blood pressure and BLL was 0.137 (*P* < 0.001); for diastolic blood pressure, the coefficient was 0.126 (*P* < 0.001) as calculated by Pearson correlation analysis. Participants with elevated blood pressure presented higher blood lead concentrations. The average BLL was 10.8 μg/dL (95% CI, 10.4–11.2 μg/dL) for participants with higher systolic pressure and 10.9 μg/dL (95% CI, 10. 6–11.3 μg/dL) for participants with higher diastolic pressure compared with those with normal systolic and diastolic blood pressure (8.5 μg/dL; 95% CI, 8.4–8.7 μg/dL) and (8.4 μg/dL; 95% CI, 8.3–8.6 μg/dL), respectively. Interestingly, we found that workers in mini-factories had the highest BLL (20.3 μg/dL; 95% CI, 19.0–21.6 μg/dL) for overall participants, while the average BLL in large factories was much lower (6.0 μg/dL; 95% CI, 5.8–6.2 μg/dL) when comparing with factories of other scales. Likewise, those employees in private factories had a higher BLL (9.6 μg/dL; 95% CI, 9.5–9.8 μg/dL), and it was much lower (4.0 μg/dL; 95%CI, 3.7–4.2 μg/dL) in state-owned factories. Participants who worked in the electrical machinery and equipment manufacturing industry had higher BLL (9.1 μg/dL; 95% CI, 9.0–9.3 μg/dL) (Table 1).

In an unadjusted analysis, hypertensive participants were older (41.3 years old, *P* < 0.001) and had a longer history of exposure (4.4 years, *P* < 0.001) than did non-hypertensive participants. The prevalence of hypertension was 34.3% for mini-factories, 17.1% for small factories, 18.6% for middle factories and 19.5% for large factories. Similar to the results of BLL, the hypertension prevalence of private factories was highest (19.9%) among the overall population (Table 2).

**Table 2:** Participant characteristics by hypertension status.

Characteristics	No Hypertension (n = 17578)	Hypertension (n = 4110)	*P* value
Age (years)	35.8	41.3	< 0.001
Exposure years	3.6	4.6	< 0.001
Blood lead (μg/dL)	8.38	10.75	< 0.001
Gender			< 0.001
Male	10069 (76.5%)	3092 (23.5%)	
Female	7509 (88.1%)	1018 (11.9%)	
Factory scale			< 0.001
mini	287 (65.7%)	150 (34.3%)	
small	2771 (82.9%)	570 (17.1%)	
middle	9083 (81.4%)	2075 (18.6%)	
large	5437 (80.5%)	1315 (19.5%)	
Economic type			< 0.001
State-owned	716 (85.2%)	124 (14.8%)	
Collectively owned	2043 (85.3%)	353 (14.7%)	
Privately owned	13403 (80.1%)	3338 (19.9%)	
Foreign-owned	1416 (82.8%)	295 (17.2%)	
Industry category			< 0.001
Electrical machinery and equipment manufacturing industry	14049 (81.0%)	3288 (19.0%)	
Computer, communications and other electronic equipment manufacturing industry	1175 (89.2%)	142 (10.8%)	
Others	2354 (77.6%)	680 (22.4%)	

The results of the linear regression models for SBP and DBP are shown in Table 3. There was a significant association of blood lead with both SBP and DBP. After adjustment, participants in quartile 4 of blood lead presented a 1.34 mmHg (95% CI, 1.15–1.53 mmHg; *P* < 0.001) average difference in SBP when compared with those in quartile 1. For DBP, the average difference was 0.70 mmHg (95% CI, 0.56–0.84 mmHg; *P* < 0.001). As shown in Table 4, the adjusted OR for hypertension comparing the highest to the lowest blood lead quartiles was 1.11 (95% CI, 1.08–1.15; *P* < 0.001).

**Table 3:** Change (95% CI) of systolic and diastolic blood pressure by blood levels (μg/dL).

	Blood lead (μg/dL)	Systolic blood pressure (mmHg)				Diastolic blood pressure (mmHg)			
				Model 1		Model 2		Model 1		Model 2	
		B (95%CI)	*P*	B (95%CI)	*P*	B (95%CI)	*P*	B (95%CI)	*P*
Overall	Quartile 1 (≤ 4.6)	Ref.		Ref.		Ref.		Ref.	
	Quartile 2 (4.7–10.9)	0.58 (0.01–1.16)	0.048	1.98 (1.37–2.59)	< 0.001	0.50 (0.08–0.91)	0.018	0.65 (0.22–1.09)	0.004
	Quartile 3 (11.0–17.5)	1.63 (1.34–1.92)	< 0.001	1.81 (1.51–2.10)	< 0.001	1.21 (1.00–1.42)	< 0.001	1.26 (1.05–1.48)	< 0.001
	Quartile 4 (> 17.5)	1.18 (1.00–1.37)	< 0.001	1.34 (1.15–1.53)	< 0.001	0.69 (0.55–0.82)	< 0.001	0.70 (0.56–0.84)	< 0.001
Male	Quartile 1 (≤ 4.6)	Ref.		Ref.		Ref.		Ref.	
	Quartile 2 (4.6–11.0)	-0.27 (-1.00–0.45)	0.458	1.80 (1.03–2.56)	< 0.001	0.16 (-0.37–0.69)	0.549	0.61 (0.04–1.18)	0.036
	Quartile 3 (11.1–16.2)	1.34 (0.96–1.73)	< 0.001	1.19 (0.80–1.59)	< 0.001	1.12 (0.84–1.14)	< 0.001	0.90 (0.60–1.19)	< 0.001
	Quartile 4 (> 16.2)	0.30 (0.03–0.56)	0.027	0.71 (0.43–0.98)	< 0.001	0.21 (0.01–0.4)	0.037	0.22 (0.01–0.43)	0.037
Female	Quartile 1 (≤ 4.6)	Ref.		Ref.		Ref.		Ref.	
	Quartile 2 (4.6–10.2)	1.92(1.07–2.77)	< 0.001	2.53(1.16–3.40)	< 0.001	0.56(0.04–1.16)	0.069	0.51(-0.11–1.13)	0.105
	Quartile 3 (10.3–16.2)	1.57(1.15–1.99)	< 0.001	1.80(1.37–2.22)	< 0.001	1.06(0.76–1.36)	< 0.001	1.12(0.82–1.42)	< 0.001
	Quartile 4 (> 16.2)	1.25(0.97–1.52)	< 0.001	1.31(1.03–1.59)	< 0.001	0.60(0.40–0.79)	< 0.001	0.52(0.32–0.72)	< 0.001

Model 1 was adjusted for gender, age (≤ 30, 31–45, ≥ 46), exposure years (< 2, 2–4, > 4). Model 2 was further adjusted for factory scale (mini, small, middle, large), economic type (state-owned, collectively owned, privately owned, foreign-owned), Industry category (electrical machinery and equipment manufacturing industry, electronic equipment manufacturing industry, others).

**Table 4:** OR (95% CI) of hypertension by blood lead quartiles (μg/dL).

	Blood lead (μg/dL)	Model 1		Model 2	
		OR (95%CI)	*P* value	OR (95%CI)	*P* value
Overall	Quartile 1 (≤ 4.6)	Ref.		Ref.	
	Quartile 2 (4.7–10.9)	0.89 (0.80–1.00)	0.045	1.06 (0.97–1.15)	0.181
	Quartile 3 (11.0–17.5)	1.13 (1.07–1.19)	< 0.001	1.14 (1.08–1.20)	< 0.001
	Quartile 4 (> 17.5)	1.11 (1.07–1.15)	< 0.001	1.11 (1.08–1.15)	< 0.001
Male	Quartile 1 (≤ 4.6)	Ref.		Ref.	
	Quartile 2 (4.6–11.0)	0.86 (0.75–0.98)	0.026	1.23 (1.11–1.36)	< 0.001
	Quartile 3 (11.1–16.2)	1.14 (1.08–1.22)	< 0.001	1.12 (1.05–1.20)	< 0.001
	Quartile 4 (> 16.2)	1.04 (0.99–1.08)	0.090	1.05 (1.01–1.10)	0.024
Female	Quartile 1 (≤ 4.6)	Ref.		Ref.	
	Quartile 2 (4.6–10.2)	0.98(0.79–1.22)	0.870	1.04(0.83–1.30)	0.756
	Quartile 3 (10.3–16.2)	1.09(0.98–1.21)	0.122	1.13(1.01–1.26)	0.029
	Quartile 4 (> 16.2)	1.12(1.05–1.19)	0.001	1.13(1.06–1.21)	< 0.001

Model 1 was adjusted for gender, age (≤ 30, 31–45, ≥ 46), exposure years (<2, 2–4, >4). Model 2 was further adjusted for factory scale (mini, small, middle, large), economic type (state-owned, collectively owned, privately owned, foreign-owned), Industry category (electrical machinery and equipment manufacturing industry, electronic equipment manufacturing industry, others).

## Discussion

In this study, we found the association of blood lead was +1.34 mmHg (*P* < 0.001) and +0.70 mmHg (*P* < 0.001) for SBP and DBP, respectively. A downward trend of lead concentration in the blood of workers who were occupationally exposed to lead has been noted in almost all developed countries in recent decades. A BLL of 40 μg/dL is accepted as safe and as minimising the health hazardous effects in Europe, according to the air quality guidelines of WHO [22]. In America, the mean BLL of children aged 6 months to 5 years was less than 5.0 mg/dL in 2016, and many children still have BLL above the current Centres for Disease Control and Prevention (CDC) “reference value” of 5.0 mg/dL [23].

The toxic actions of lead on the human body have been relatively well studied. Prolonged exposure may lead to chronic poisoning, including disorders of haematopoiesis and of the central nervous, renal, gastrointestinal, immune and cardiovascular systems. The effects of lead on blood pressure become of increasing concern. In addition to the above toxic effects, environmental and occupational exposure to lead may cause of male infertility by reducing sperm count motility, viability, normal forms and detectable levels[24]. Lead exposure was also associated with reproductive hormone levels in Chinese males and postmenopausal females[25].

Numerous studies have sought to investigate the relationship between lead and clinical cardiovascular disease in the general population. In the National Health and Nutrition Examination Survey III Mortality Follow-up Study, which recruited 13,946 adult participants from 1988 to 1994, the hazard ratios for comparisons of participants in the highest tertile of BLL (≥ 3.62 μg/dL) with those in the lowest tertile (≥ 1.94 μg/dL) was 1.55 for cardiovascular mortality. After multivariate adjustment, the hazard ratio (95% CI) for a 3.4-fold increase in BLL was 1.78 and 1.59 for myocardial infarction and stroke mortality, respectively[26]. A study about the association of blood pressure and hypertension with blood lead found that blacks had higher SDP and DBP and higher hypertension prevalence compared with non-blacks. Blood lead was lower in whites than in non-whites and in females than in males [27].

Faramawi *et al*. [28]documented a positive linear relationship between environmental lead exposure and systolic blood pressure. When compared with the general population, the occupational population is exposed to a higher concentration of lead in the workplace. Occupational exposure to lead can promote atherosclerosis, particularly in highly exposed workers[15]. Orawan *et al*. [29] reported that there were significant correlations between the BLL of bus drivers in Thailand and blood pressure after controlling for age, BMI, alcohol consumption, smoking and physical exercise. An assessment of lead exposure risk found that the mean tibia bone lead concentration was 13.1 μg/g higher in locksmiths than in their matched controls; this difference means an OR of increased hypertension in locksmiths of between 1.1 and 2.3, based on the published literature [30]. Lead toxicity is still persistent in battery manufacture workers. The absorption of lead was more in these workers, which adversely affected blood pressure and disturbed calcium and phosphorus metabolism; this further impaired mineralisation of bone results in decreased bone mineral density [31].

Jomova *et al*. have reported that lead may be involved in oxidative stress to induce changes in the vascular endothelium. Lead could oxidise nitric oxide (NO), which is a strong endogenous factor regulating the relaxation of the vascular smooth muscle. This process leads to the formation of the highly reactive peroxynitric acid anion, which is associated with hypertension, DNA damage and increase of lipid peroxidation [32]. Oxidative stress could promote inflammation, fibrosis, and apoptosis by activating NF-κB[33]. Pharmacological inhibition of NF-κB activation has been shown to attenuate renal interstitial inflammation and decrease arterial pressure in hypertensive animals. Ramesh *et al*. have demonstrated that lead exposure induces the activation of NF-κB [34]. Shen *et al*. [35] have indicated that environmental and occupational lead exposures induced increases in PI3K-Akt signalling and partially underlie the increased blood-retinal-barrier permeability. They suggested that lead exposure may be the risk factor for increased blood-retinal-barrier permeability in diseases such as age-related macular degeneration, diabetes and stroke.

We detected the BLL of 21,688 workers who were exposed to lead in Jiangsu province. There were some highlights in our study. One of the highlights was that the number of the subjects included into this study was large enough for regression analysis. The data could represent the overall lead exposure level of workers in Jiangsu province. In addition, this study considered the factory scale, economic type and industry category, allowing for further study. Our results showed that the BLL was related to the scale of factories and the economic type. The potential cause may be that small private factories have poor prevention measures and management oversight, while large state-owned factories always have more advanced technology and formal management practices, such as periodic physical examinations. However, there were several limitations in the present study. First, some potential risk factors such as smoking and drinking status, body mass index, heart rate, liver function, and use of antihypertensive drugs were not collected. In addition, the lead levels in bones could not be estimated in our study. Measurement of bone lead concentration is considered an important and widely accepted technique of determining the long-term toxicity of lead and a more reliable measurement of cumulative lead exposure than blood lead concentration[36].

## Conclusions

Our study provided evidence on the association between lead exposure and blood pressure in an occupational population. The association raises some public health implications. First, current standards for blood lead must be lowered and a criterion for screening elevated lead exposure needs to be established in the occupational population. Additionally, risk assessment and economic analyses of lead exposure impact must cover the effect of lead. Finally, advanced management tools and public health interventions must be developed and adopted to further reduce and prevent lead exposure.

## Supporting information

S1 Table Raw data of blood lead levels and subject characteristics. (SAV)
